# Unveiling the Economic Toll of Surgical Learning Curve in Elderly Hip Fractures

**DOI:** 10.3390/jcm12154880

**Published:** 2023-07-25

**Authors:** Eyal Yaacobi, Tal Shachar, David Segal, Altaieb Agabaria, Golan Halima, Omer Marom, Nissim Ohana

**Affiliations:** 1 Department of Orthopaedic Surgery, Meir Medical Center, Tschernihovski 59 Street, Kfar-Saba 4428164, Israel; yaacobi.eyal@gmail.com (E.Y.); dudisegal@gmail.com (D.S.); marom.omer@gmail.com (O.M.); 2 Faculty of Medicine, Ramat Aviv, Tel Aviv 69978, Israel; 3 Surgical Service Unit, Meir Medical Center, Tschernihovski 59 Street, Kfar-Saba 4428164, Israel

**Keywords:** hip fractures, resident training, surgery, outcomes, implant price, implant waste

## Abstract

Can the financial impact of implant choice during the learning curve of inexperienced surgeons in hip fracture surgery be quantified? Hip fractures in the elderly are a significant medical concern, often requiring surgical interventions performed by orthopedic surgery residents. As healthcare costs rise, exploring cost reduction opportunities within the healthcare system becomes crucial. In this prospective analysis, we examined the financial implications of implant choices encountered by residents during their learning curve in hip fracture surgery. Our study included 278 surgically treated pertrochanteric fractures using the same locking cephalomedullary nail. Data on patients, surgeons (including their experience and seniority), and all implants charged by the hospital were collected. This encompassed documentation of any nail-related equipment that was opened on the operating table and whether it was subsequently used by the end of the procedure. By calculating the number and cost of these implants, we assessed the financial burden associated with suboptimal choices made during the learning curve. Our findings revealed that in 16.18% of surgeries, instances of suboptimal implant utilization occurred, highlighting the complexities of the learning process. Importantly, the rate of these challenges was not influenced by surgeon seniority or patient characteristics. The mean additional cost per surgery was determined to be USD 65.69 ± 157.63 for surgeries with suboptimal implant utilization, compared to USD 56.55 ± 139.13 for surgeries without such challenges. Although there was a trend towards higher implant-related costs in resident-led surgeries, the difference did not reach statistical significance. These findings underscore the feasibility of enabling residents to autonomously perform intramedullary nailing surgeries, even without specialist supervision, while incurring minimal additional expenses during the learning curve. By acknowledging the financial implications associated with the learning curve in the management of hip fractures, we can strive to optimize healthcare costs, thus addressing an important aspect of this issue.

## 1. Introduction

Hip fractures impose significant healthcare expenses as well as high mortality, morbidity, and reoperation rates [1]. In the United States alone, approximately 310,000 individuals were hospitalized with hip fractures in 2003 [2], and it is projected that the global incidence of hip fractures will surpass 6 million by 2050 [3]. The annual healthcare costs associated with hip fractures in the United States range from 10.3 to 15.2 billion dollars [4]. Hip fracture surgery is a prevalent procedure within the field of orthopedic surgery, often performed on elderly patients [5].

Orthopedic training programs employ an apprenticeship model, wherein surgical residents learn and progressively gain proficiency in various procedures under supervision. Consequently, hip fracture surgery stands as one of the basic surgeries that residents autonomously perform in the operating room (OR), following a learning curve trajectory [6,7,8]. However, this increasing autonomy may lead to extended operative durations, elevated surgical complication rates, and suboptimal utilization of resources [9,10].

Effective OR procedures necessitate the appropriate selection and usage of medical devices, with the United States spending over 150 billion dollars annually on theis equipment [11]. Implant costs constitute a substantial portion of the OR budget, with surgical implants often representing the primary contributor to orthopedic surgery expenses, accounting for up to 87 percent of the overall cost [12]. Reducing implant costs and optimizing resource utilization are vital aspects of cost containment strategies in orthopedic trauma surgery. Although physicians are encouraged to consider cost factors in device selection, limited awareness of cost rates persists among them [13,14,15]. Several studies have attempted to quantify the frequency and cost of unused surgical implants. For instance, Zymiel et al. reported a modest yet noteworthy annual incidence of 2% for intraoperative waste of hip and knee arthroplasty implants [6,16]. Conversely, Bosco et al. observed a 30% incidence of implant waste in trauma surgeries [17].

Previous investigations have indicated that less experienced surgeons exhibit a higher propensity for unused implants compared to their more experienced counterparts [18]. However, little research has focused on assessing the financial implications of unused implants resulting from the learning curve of surgical residents. Therefore, the objective of this study was to evaluate the additional financial burden attributable to suboptimal implant choices made by surgical residents during their training years. This research holds particular significance due to the prevalence of hip fractures in the elderly population, with orthopedic residents widely involved in their treatment worldwide. Given the imperative to reduce healthcare costs, identifying and quantifying the financial burden associated with the learning curve can inform strategies for optimizing resource utilization and enhancing the overall efficiency and quality of patient care. By documenting the surgical procedures, surgeons’ seniority, implant utilization, and associated costs, our study aims to provide valuable insights into the financial implications of the learning curve in orthopedic surgery. This research holds significance not only in terms of optimizing resource allocation but also in enhancing the overall quality and efficiency of patient care.

## 2. Methods

Following the approval of our institutional review board, we conducted a comprehensive retrospective analysis to investigate the surgical management of near-trochanteric femur fractures. This study encompassed both closed and open reduction procedures, utilizing internal fixation with the Gamma3 hip nail (Stryker Corporation, Kalamazoo, MI, USA).

The data collection period spanned from 1 January 2021, to 31 December 2021, and focused exclusively on surgeries performed with a single surgical fixation system in a single medical institute.

The primary aim was to evaluate the impact of surgeon experience on implant utilization and related costs. To achieve this, we meticulously retrieved data on elderly patients who were urgently admitted due to acute hip fractures to our department during the study period. Only patients with intertrochanteric or sub-trochanteric fractures requiring nailing were included in the study. The level of the surgeon’s seniority and the implants that were opened during the procedure, regardless of their usage by the end of the procedure, were recorded. The implant cost of surgery for each patient was retrieved from hospital bills. These data were matched with the actual use that was recorded during surgery. Any supplementary material that was added to the bill of a native nailing system was defined as “wasted” implants and calculated.

The surgical team was divided into two distinct groups: “young” orthopedic surgery specialists, who had completed 1 to 3 years post-residency, and 5th and 6th-year orthopedic surgery residents under the supervision of un-scrubbed senior surgeons of equivalent seniority.

Throughout the study, our focus was on recording all non-reusable implants utilized during the surgeries. This included implants that required replacement during the course of the procedure. The reasons for implant replacement were meticulously extracted from the comprehensive patient records, providing us with valuable insights into the factors contributing to suboptimal implant choices. Among the reasons identified, three distinct categories emerged: Firstly, instances of breaking sterility during the surgical procedure were noted as a factor leading to implant replacement. Secondly, measurement errors pertaining to the proximal lag or locking screws were observed, highlighting instances where inaccuracies in measurements resulted in the need for implant replacement. Finally, variations in nail length were identified, with some cases requiring a longer or shorter nail than the initially planned implant size. These three categories encompass the primary factors contributing to suboptimal implant choices, emphasizing the significance of maintaining sterility, ensuring precise measurements, and accurately selecting the appropriate implant length to avoid the need for subsequent replacements. To establish a comprehensive cost analysis, we obtained the current prices of each implant component from the medical institute’s perspective as of December 2022. Specifically, we collected the costs associated with the intramedullary nail (short = USD 395, long = USD 537), proximal lag screw (USD 199), set screw (USD 114), and distal cortical screw (USD 87). These figures were based on the prevailing rates within the authors’ country.

The additional cost incurred due to the utilization of extra implant components was calculated by dividing the mean cost of the additional implant parts used in all surgeries by the price of the basic implant set, which included the intramedullary nail, lag screw, set screw, and cortical screw. It is important to note that surgeries where no additional implants were required were also included in the calculation, with the additional cost of these procedures being considered “0”.

## 3. Statistical Analysis

Statistical analysis was performed using SPSS 28.0 software (IBM, Armonk, NY, USA). Descriptive statistics were utilized to present the raw data. Categorical variables were compared using the Fisher’s exact test, while continuous variables were compared using the Mann–Whitney U test. The multiplier, which represents the additional cost incurred due to the utilization of extra implant components, was presented as the mean ± standard deviation (SD). Statistical significance was defined based on a power of 0.8 and a beta of 0.05. These criteria were used to assess the significance of the findings derived from the statistical analysis.

## 4. Results

**Figure 1:**
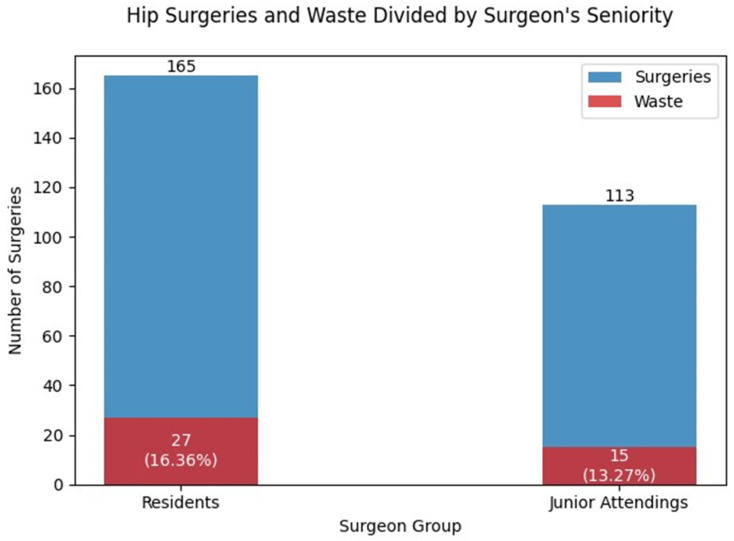
Surgeries represent the number of surgeries performed by surgeons based on seniority, while Waste indicates the percentage of surgeries with observed implant waste in each surgeon group. The differences are not significant (*p* > 0.05).

A total of 278 cases were included in the analysis, with 165 (59.36%) surgeries performed by residents and 113 (40.64%) surgeries performed by senior surgeons. Figure 1 shows the deviation between the two groups of surgeons: residents and junior attendings. Among these cases, 42 (15%) surgeries were documented as having experienced an unforeseen outcome resulting in implant adjustments. The occurrence of these adjustments was observed in 27 (16.36%) surgeries led by residents and 15 (13.27%) surgeries led by junior attendings, with no statistically significant difference between the two groups (*p* = 1).

**Figure 2:**
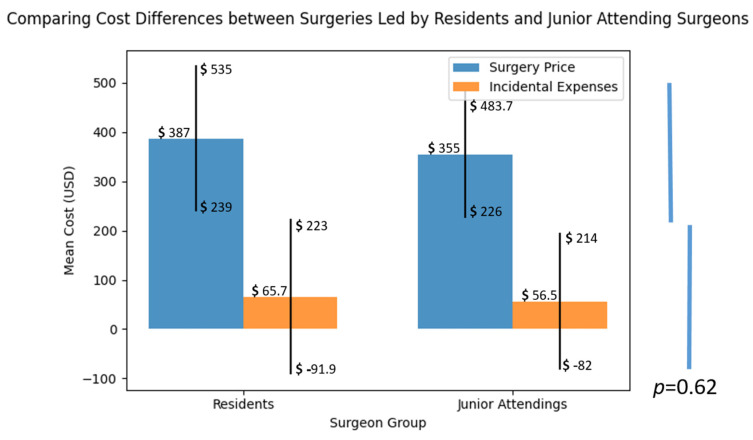
Surgery Price represents the mean cost of surgeries performed by residents and junior attending surgeons, while incidental expenses indicate the mean additional expenses associated with those surgeries. The differences are not significant (*p* > 0.05).

The mean cost of these intra-operative adjustments was found to be USD 387.09 ± 148.05 per occurrence in the resident group and USD 355.04 ± 128.76 per occurrence in the junior attending group, with no statistically significant difference observed (Figure 2). When considering all surgeries, including those that required implant adjustments, the mean cost per surgery associated with these events was USD 65.69 ± 157.63, compared with USD 56.55 ± 139.13 for surgeries without such events, again with no significant difference (*p* = 0.62).

**Figure 3:**
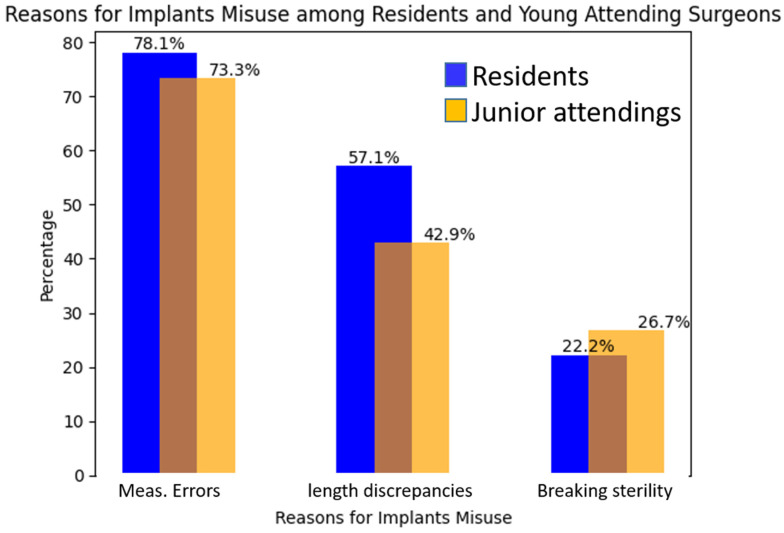
Comparison of the different reasons for implant misuse in the two groups of surgeons, residents and junior attendings. The differences are not statistically significant.

Figure 3 provides the causes of the implant’s extra costs, broken down by the two groups of surgeons and divided by the types of surgeries. Throughout the study period, closed reduction and internal fixation (CRIF) accounted for 83.3% of the surgeries, while open reduction and internal fixation (ORIF) represents the remaining 16.7%. It should be emphasized that we refer to ORIF as a procedure that was performed intentionally and not as a conversion from CRIF.

Additionally, we identified instances where residents made alternative implant selections, specifically starting with a short nail and replacing it with a long nail, which occurred in 12 cases operated by residents compared to only 4 performed by junior attending surgeons (Figure 3).

Figure 3 presents the reasons for implant misuse. The most common cause of implant adjustments was attributed to measurement errors, accounting for 78.1% among residents and 73.3% among young attending surgeons. Following this, length discrepancies (longer nails vs. shorter nails) played a contributing role, as observed in 57.1% of cases for residents and 42.9% for young attending surgeons. The least common cause for both groups was related to factors such as maintaining a sterile environment, with rates of 26.7% for young attending surgeons and 22.2% for residents.

## 5. Discussion

Medical residents play an integral and multifaceted role within the healthcare system, contributing significantly to patient care, medical education, and research [19]. In the realm of surgery, residency training assumes even greater significance as it offers aspiring surgeons a unique opportunity to acquire invaluable hands-on experience, refine their technical skills, and cultivate expertise in their chosen specialty. The comprehensive surgical experience gained during residency serves as a pivotal phase in the development of proficiency in performing intricate procedures, allowing residents to navigate the intricacies of surgery with increasing confidence and competence.

The hands-on nature of residency training enables residents to actively participate in a wide range of surgical procedures, providing them with exposure to diverse patient cases and complex scenarios. This exposure fosters the cultivation of essential technical skills, including precise suturing techniques, proficient use of surgical instruments, and the ability to effectively manage intraoperative challenges. Through repeated practice and guidance from experienced mentors, residents gradually refine their technical abilities, allowing them to perform procedures with greater precision and efficiency.

Equally important, residency training enhances residents’ decision-making skills, a crucial aspect of surgical care [20]. The exposure to a myriad of patient presentations and surgical scenarios enables residents to develop the ability to make critical judgments in a time-sensitive and high-pressure environment. They learn to assess risks, evaluate treatment options, and make informed decisions that prioritize patient safety and optimize surgical outcomes. This development of sound decision-making skills is vital for surgeons, as it directly impacts the quality of care delivered to patients.

Moreover, residency training offers a platform for residents to actively engage in medical education and research. Residents often participate in educational activities, such as grand rounds, case presentations, and surgical conferences, where they can exchange knowledge and learn from their peers and experienced faculty members. Additionally, residents have the opportunity to contribute to ongoing research projects, assisting in expanding the boundaries of medical knowledge and driving advancements in surgical techniques and patient care. In this study, we focused on examining whether surgical residents’ autonomy in the operating room leads to a financial burden on the healthcare facility. Autonomy refers to the level of independence granted to residents when performing surgical procedures. While resident autonomy is crucial for their professional growth and acquisition of skills, concerns have been raised regarding potential negative consequences, such as increased costs due to variations in practice patterns. Our study delves into the economic impact of hip fractures in the elderly, with a specific focus on the costs associated with the surgical implant component. While there are various expenses linked to hip fractures, such as hospitalization, surgical procedures, rehabilitation services, and ongoing medical care, our study primarily examines the financial implications related to the surgical implant. By narrowing our focus to this specific aspect, we aim to shed light on the economic burden directly tied to the implant, recognizing that it represents a significant but distinct portion of the total expenses incurred. Understanding the financial impact of the surgical implant can inform healthcare providers, policymakers, and stakeholders in developing strategies to optimize cost-effective approaches without compromising patient outcomes.

The findings in this study revealed that residents had more cases of intra-operative implant changes compared to junior attending surgeons (Figure 1). However, it is important to note that these differences were not found to be statistically significant. These results are consistent with other reports in the literature [21,22]. One potential explanation could be attributed to the learning curve associated with residents’ training, which may initially require using more implants for intra-operative adjustments. Evidence supporting the benefit of surgical experience can be found in the research conducted by Mabry et al. [6], who compared the outcomes of surgical treatment for subcapital fractures of the hip joint between specialists who had completed a Fellowship and specialized in general orthopedics. Their study demonstrated a distinct advantage for experienced surgeons, resulting in more successful surgical outcomes. However, it is essential to qualify the conclusions of this study, as they differ in terms of the complexity and potential complications of the femoral neck nailing procedure investigated in our study. 

The study findings revealed that measurement variation, among other factors such as sterility and length discrepancies, contributed to the need for implant change in elderly hip fractures. Notably, measurement variation was found to be the primary factor, accounting for a significant portion of cases requiring implant changes. Specifically, measurement variation accounted for 78.1% of the instances, indicating its substantial impact. This highlights the importance of ensuring precise and accurate measurements during surgical procedures, particularly in complex cases like hip fractures in the elderly population. Efforts to address and minimize measurement variation, along with addressing other contributing factors, can potentially reduce implant waste and improve patient outcomes in orthopedic surgeries (Figure 3). We assessed the inclination of both residents and junior attendings towards performing open reduction and internal fixation (ORIF) or closed reduction and internal fixation (CRIF) for hip fractures and found no differences between the groups. Our study’s findings show that there is no statistical relationship between the types of fracture reduction and the increased unnecessary use of implants. Our findings echo the literature [23] and highlight the importance of continuous quality improvement initiatives and training interventions to minimize discrepancies and enhance patient safety. Shorter to longer nail replacement and vice versa, occurring more often in cases operated by residents, emphasizes the need for ongoing education and guidance to improve decision-making skills and optimize implant selection among residents.

While it is well established that residents are more prone to measurement variations in practice compared to more experienced surgeons [24], it is essential to recognize that this does not imply that all residents exhibit similar patterns or that all junior attendings are immune to variations. Variability in skill levels and experience exists across surgeons at different stages of training [10]. Moreover, the learning process involves a period of supervised practice, during which residents refine their abilities and gradually reduce practice variations. As residents gain experience, their surgical skills, decision-making abilities, and communication skills improve, eventually approaching the level of proficiency seen in senior surgeons [24].

It is crucial to recognize that variations in practice patterns and decision-making skills are not unique to residents but exist across surgeons at different stages of training [20]. As residents gain experience, their surgical skills, decision-making abilities, and communication skills improve, approaching the level of proficiency seen in senior surgeons. Future research should focus on optimizing resource utilization and reducing practice variations to improve the cost-effectiveness of orthopedic surgical procedures.

Although there are limited publications specifically addressing surgical implant utilization in the current literature [6], existing studies suggest that suboptimal resource utilization significantly contributes to the cost of orthopedic surgeries [17]. However, these studies often fail to explore the underlying reasons behind these variations or consider the level of training of the surgeons involved. Our study aims to bridge this gap by investigating the impact of residents’ autonomy on implant utilization and associated costs. By shedding light on this issue, we hope to stimulate further research and encourage the development of strategies to optimize resource utilization, thereby improving the cost-effectiveness of orthopedic surgical procedures.

While our study provides valuable insights, it is important to acknowledge its limitations. First, the study was conducted at a single center, which may limit the generalizability of the findings to other healthcare settings. Further multi-center studies are needed to validate our results across diverse patient populations and surgical environments. Second, the sample size in our study was relatively small, which may limit the statistical power of the analysis and the ability to detect subtle differences. Future studies with larger sample sizes would provide more robust evidence. Lastly, our study focused on short-term outcomes and did not explore long-term surgical outcomes, which could provide a more comprehensive understanding of the clinical and financial implications of these surgeries.

The autonomy granted to surgical residents in the operating room is essential for their professional growth and skill development. While residents may exhibit variations in practice patterns, including implant utilization, our study shows that the financial burden associated with increased implant utilization by residents was not significantly different when considering the overall costs. The primary factor contributing to implant changes was measurement variation, which occurred more frequently among residents. This highlights the importance of continuous quality improvement initiatives and training interventions to minimize discrepancies and enhance patient safety.

These findings shed light on the specific types of surgeries and causes of implant adjustments encountered in hip fracture cases, providing valuable insights into areas where improvements in surgical technique and training can be targeted to minimize intraoperative adjustments and optimize patient outcomes.

In summary, surgical residents’ autonomy in the operating room is a crucial component of their training and professional development. Understanding the impact of this autonomy on implant utilization and associated costs can inform strategies to optimize resource utilization and improve the cost-effectiveness of orthopedic surgical procedures. Continuous quality improvement initiatives and ongoing education are essential to minimize variations, enhance patient safety, and ensure the delivery of high-quality surgical care.
